# Supplementary material for: Predicting environmental stressor levels with machine learning: a comparison between amplicon sequencing, metagenomics, and total RNA sequencing based on taxonomically assigned data
Source: Front Microbiol. 2023 Nov 24;14:1217750. doi: 10.3389/fmicb.2023.1217750 (PMC10704153; doi:10.3389/fmicb.2023.1217750)
Supplement: Supplementary file 4 [file Data_Sheet_4.docx]

# Supplemental Figures


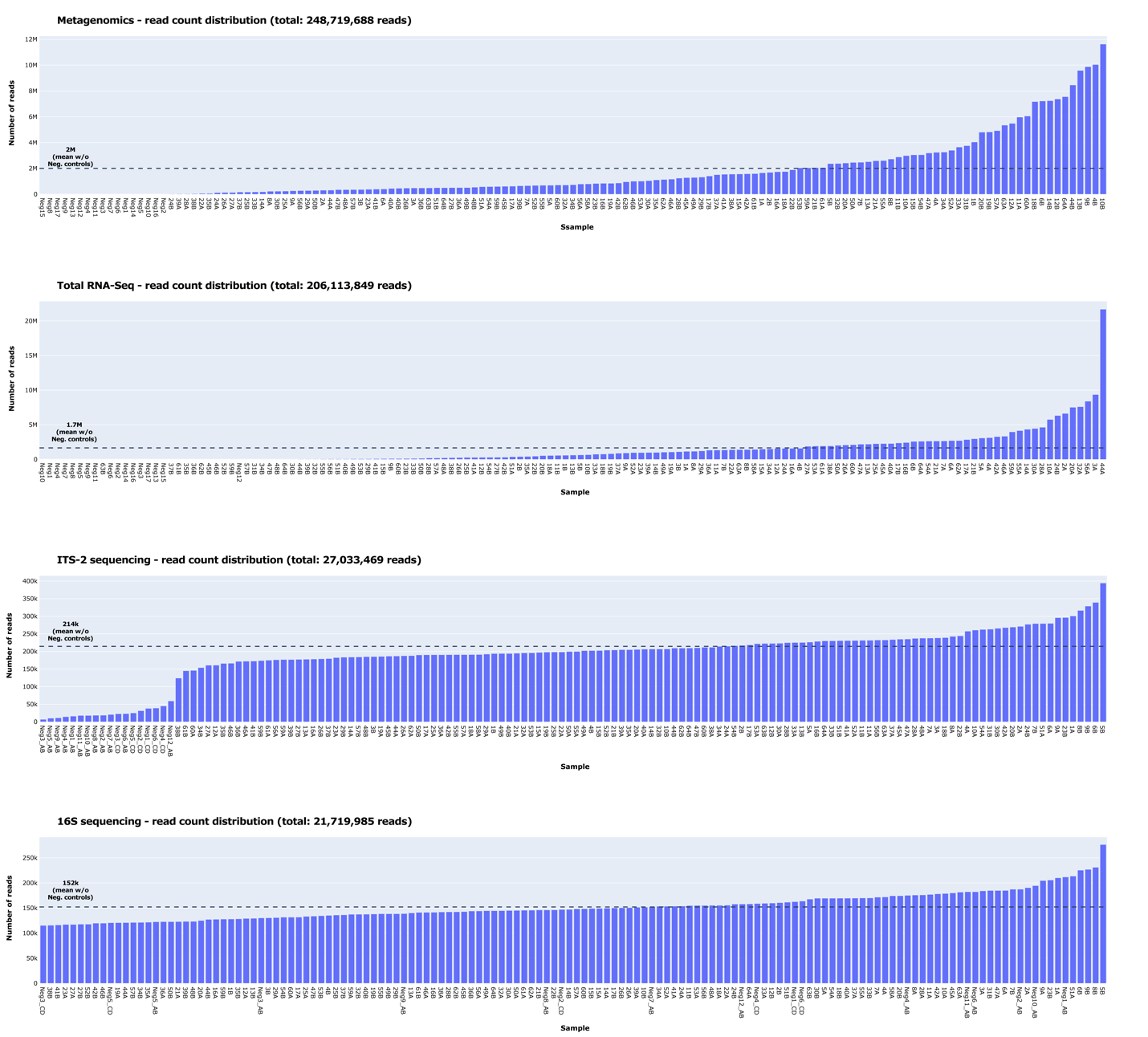


Supplementary Figure S1: Number of reads and read distribution obtained for metagenomics, total RNA-Seq, 16S sequencing, and ITS-2 sequencing. Mean average numbers of reads (excluding negative controls) are indicated by dashed lines. Note that the y-axis range differs among graphs. The number of reads received per metagenomics and total RNA-Seq sample varied substantially since libraries were normalized based on volume rather than concentration so that the relative number of reads per sample mirrored the relative amount of DNA/RNA, avoiding an over- or underrepresentation of samples with higher or lower amounts of DNA/RNA.


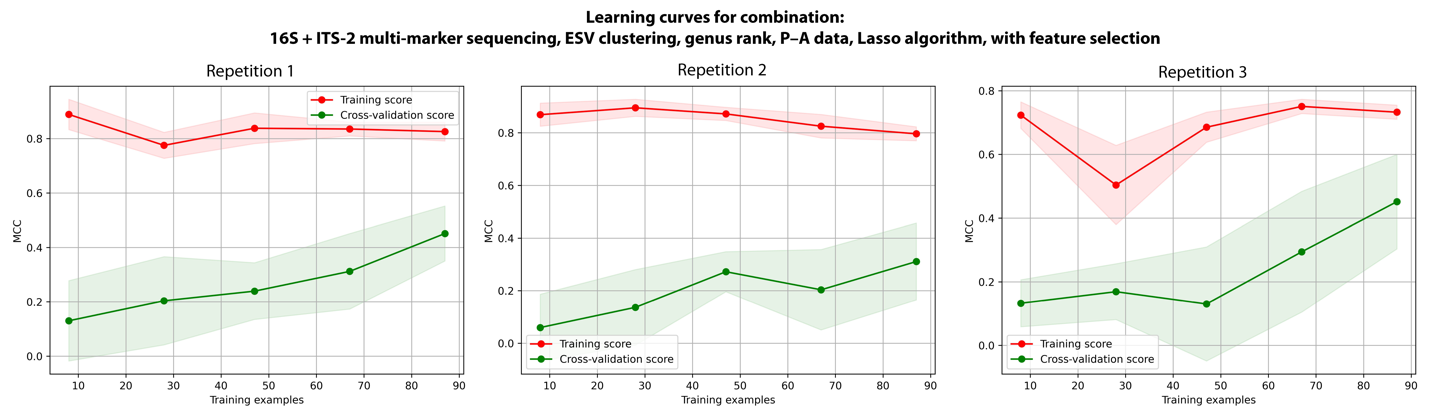


Supplementary Figure S2: Learning curves generated during model training of the best-performing combination of sequencing and data-processing methods across three repetitions. The constant increase in the cross-validation score indicates that the models were underfitted.

# Supplemental material – setup of the SSU and LSU SILVA kraken2 database

We downloaded the available SILVA132_NR99 SSU and LSU databases and merged them into one SILVA database, removing duplicates that falsely occurred in both databases according to the SILVA-arb support. We standardized the taxonomy nomenclature of the SILVA database by translating the taxonomy of all SILVA reference sequences into the NCBI Genbank taxonomy before building SILVA reference databases. Note that some SILVA reference sequences are annotated with species names that do not match the rest of their taxonomic annotation. To translate the SILVA taxonomy into Genbank taxonomy, we first made sure that the last taxonomic level of each SILVA reference sequence was a species name that fit the remaining taxonomy. Therefore, we checked if the genus part of the species name matched the genus name for each SILVA reference sequence, and if not, the species name was converted to *NA*. Then, we checked each taxonomic level of each SILVA reference sequence for matches among first scientific and then non-scientific names in the Genbank taxonomy file names.dmp (available through the NCBI archive, part of taxdmp.zip, <https://ftp.ncbi.nlm.nih.gov/pub/taxonomy/>), beginning at the last level, and if a match was found, the respective Genbank taxonomic ID was used for that SILVA reference sequence. If no match was found for that level, the process was repeated for the next higher level until a match was found. If no match was found overall, the reference sequence was assigned with *NA*. Note that we removed all Genbank names containing “environmental”, “uncultured”, “unidentified”, and “metagenome” from the names.dmp file prior to translation to exclude them from the matching process.

The kraken2 database was set up following the developer’s instructions (<https://github.com/DerrickWood/kraken2/blob/master/docs/MANUAL.markdown>). To set up the kraken2 database for the merged SSU and LSU SILVA database, we translated the taxonomy of each reference sequence into Genbank taxonomy IDs as described above, added the IDs to each reference sequence, and created files in the same format as the taxonomy files of the Genbank database, names.dmp and nodes.dmp (part of taxdmp.zip, <https://ftp.ncbi.nlm.nih.gov/pub/taxonomy/>). We automated the entire process with a script that is available on GitHub (<https://github.com/hempelc/metagenomics-vs-totalRNASeq>), which incorporates the SILVA taxonomy files taxmap_slv_ssu_ref_nr_138.1.txt.gz, taxmap_slv_lsu_ref_nr_138.1.txt.gz, and tax_slv_ssu_138.1.txt.gz available through the SILVA archive (<https://www.arb-silva.de/download/archive/>).
